# Natural language processing pipelines to annotate BioC collections with an application to the NCBI disease corpus

**DOI:** 10.1093/database/bau056

**Published:** 2014-06-15

**Authors:** Donald C. Comeau, Haibin Liu, Rezarta Islamaj Doğan, W. John Wilbur

**Affiliations:** National Center for Biotechnology Information, National Library of Medicine, National Institutes of Health, 8600 Rockville Pike, Bethesda, MD 20894, USA

## Abstract

BioC is a new format and associated code libraries for sharing text and annotations. We have implemented BioC natural language preprocessing pipelines in two popular programming languages: C++ and Java. The current implementations interface with the well-known MedPost and Stanford natural language processing tool sets. The pipeline functionality includes sentence segmentation, tokenization, part-of-speech tagging, lemmatization and sentence parsing. These pipelines can be easily integrated along with other BioC programs into any BioC compliant text mining systems. As an application, we converted the NCBI disease corpus to BioC format, and the pipelines have successfully run on this corpus to demonstrate their functionality. Code and data can be downloaded from http://bioc.sourceforge.net.

**Database URL:** http://bioc.sourceforge.net

## Introduction

The BioCreative IV Interoperability track (1) addressed the goal of interoperability—a major barrier for wide-scale adoption of available text mining tools. BioC (2), a new format and associated source code libraries for sharing text and annotations, allows for the simple and convenient processing of text corpora. With the provided libraries, it is straightforward to incorporate BioC code into existing programs to read in data from BioC formatted input files and write out results to BioC formatted output files. As part of this track, the community contributed BioC-formatted data sets and BioC-compliant tools for various useful biomedical natural language processing (NLP) tasks.

Our contributions to the interoperability track of the BioCreative IV challenge are BioC text-preprocessing pipelines in C++ and Java. Text preprocessing is integral to virtually all NLP systems. It processes the original text into meaningful units that contain important linguistic features before performing subsequent text mining strategies. Poor text preprocessing performance will have a detrimental effect on downstream processing. Generally, several preprocessing steps need to be performed, such as sentence segmentation, part-of-speech (POS) tagging and sentence parsing.

The BioC NLP pipeline tools are an adaptation of state-of-the-art text preprocessing tools and produce corresponding text analyses in the BioC format. The tools are widely used in the target domain and have been reported to yield competitive results on biomedical texts (3–8). The intention of our work is to provide essential text preprocessing functionalities to BioC users. The pipelines are implemented in a flexible way so that users can incorporate other tools according to their needs.

To test and verify the BioC implementations of the pipelines, we used the NCBI disease corpus (9, 10) as a model corpus. This corpus was recently developed by a team of 14 annotators and comprises 793 PubMed abstracts, manually annotated for disease mentions and corresponding Medical Subject Headings (MeSH^®^) or Online Mendelian Inheritance in Man^®^ (OMIM^®^) concepts. This resource can be used for developing machine learning methods for disease name recognition and normalization [see for example (11)], and so it is an appropriate corpus to illustrate the features of NLP (because machine learning methods for named entity recognition use linguistic features to build their models).

Another notable example of the usefulness of the BioC pipeline tools is their application in the BioNLP 2013 shared task on event extraction (12). The C++ pipeline was one of the tools that provided preprocessing results for all subtasks of the shared task. These data and codes were used successfully by three separate teams during the challenge (13–15) and are still available to the public through the BioNLP shared task Web site.

The outputs of the BioC NLP pipeline programs provide examples of how the BioC format links the results of different tools in an interoperable and integrated fashion. This demonstrates how different programs can use and produce data in a consistent format, regardless of their implementation language. The BioC NLP pipelines are freely available to the research community and are released as open-source software that can be downloaded (http://bioc.sourceforge.net).

## Methods

BioC code was released in 2013 in C++ and Java. Based on this BioC code, we created NLP pipelines in both languages as part of the BioCreative IV Interoperability Track. In this section, we describe the text preprocessing tools that make up the NLP pipelines. We explain all the tools individually and how they use the BioC environment to work and interoperate together.

### C++ pipeline

**Figure 1.**
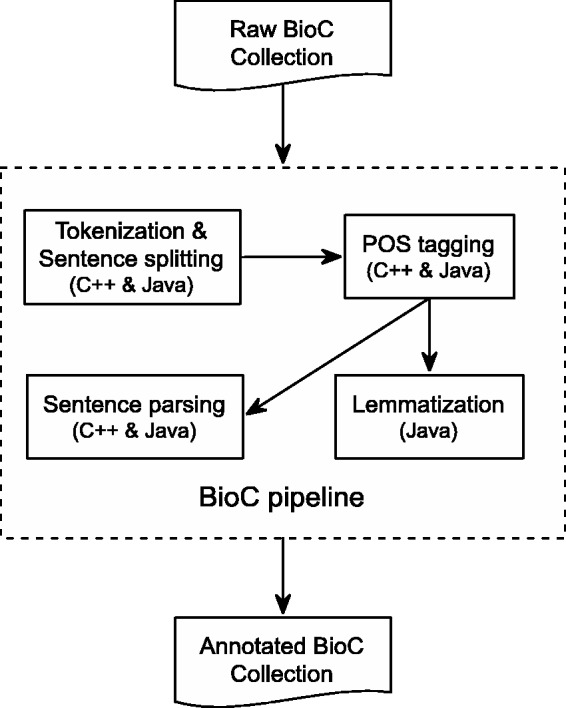
BioC Text-preprocessing Pipeline. All components are available in both C++ and Java, except for Lemmatization, which is only available in Java.

C++ is a high-performance compiled language with good execution time and memory usage. Figure 1 shows the overall flow of our pipeline. The different tools, sentence segmentation, tokenization, POS tagging and dependency parsing are implemented as stand-alone programs. They are represented by the inner boxes in Figure 1. This is convenient if the results of only one, or a few, of the tools are desired.

The components of the C++ pipeline were drawn from the MedPost (16) collection of NLP tools. One challenge was that MedPost normalizes some of the results. For example, multiple spaces between tokens in a sentence would be normalized to a single space. This had to be taken into account when determining the offset to the original text as encouraged by the BioC format.

#### Sentence segmenting and tokenizing

Tokenization is an important component of biomedical language processing. MedPost uses an efficient rule-based approach for sentence and token segmentation. In support of our choice of MedPost as a reasonable library for our BioC implementation, a recent paper reported that MedPost achieved the best performance for biomedical tokenization among several different tools and methods (17).

#### Parts-of-speech

The principle MedPost tool is a high accuracy POS tagger trained on a MEDLINE corpus (16). Using its own tag set, it achieves 97.43% accuracy on 1000 test sentences sampled from MEDLINE. It achieves 96.9% accuracy using the Penn Treebank tag set. It has been widely used (18–25) and is included in the popular LingPipe natural language toolkit (http://alias-i.com/lingpipe/).

#### Dependency parse

The C++ pipeline also includes a wrapping of the C&C dependency parser (26) developed by the MedPost developer. As this parser uses Briscoe & Carroll-style grammatical relations, in addition to the expected head and dependent tokens, some grammatical relations include a type token (http://www.sussex.ac.uk/Users/johnca/grde scription/index.html). Any initial_gr information is preserved in an infon. This is described in the cnc.key file.

### Java pipeline

Figure 1 also represents the detailed annotation flow of the Java preprocessing pipeline, which includes text tokenization, sentence segmenting, POS tagging, lemmatization and sentence parsing. The pipeline takes as input a BioC collection. Preprocessing is then invoked for each BioC passage on which the integrated tools are performed sequentially to produce corresponding text analyses. In the end, the generated annotations, along with the BioC collection information, are inserted into a BioC data structure to produce an annotated BioC file.

#### Sentence segmenter

An efficient sentence segmenter, DocumentPreprocessor, is used to produce a list of sentences from a plain text. It is a creation of the Stanford NLP group using a heuristic finite-state machine that assumes the sentence ending is always signaled by a fixed set of characters. Tokenization is performed by the default rule-based tokenizer of the sentence segmenter, PTBTokenizer, before the segmenting process to divide text into a sequence of tokens. The ‘invertible’ option of the tokenizer is invoked to ensure that multiple whitespaces are reflected in token offsets so that the resulting tokens can be faithfully converted back to the original text. Sentence segmentation is then a deterministic consequence of tokenization.

#### POS tagging

The MaxentTagger based on a maximum entropy model is used for POS tagging. The MaxentTagger is the default POS tagger used by the Stanford parser before parsing the text.

#### Lemmatization

BioLemmatizer (27) is used to perform the morphological analysis of biomedical literature. It has been demonstrated that the BioLemmatizer achieves the best lemmatization performance on biomedical texts and contributes to biomedical information retrieval/extraction tasks. The word form and the POS of a token are required as input to the BioLemmatizer to retrieve the corresponding lemma. A recent enhancement to BioLemmatizer is the ability to normalize British and American spellings. This addresses another area where surface differences conceal the same concept.

#### Sentence parsing

The POS-tagged sentences are then submitted to the Stanford unlexicalized natural language parser (28) to analyze the syntactic and semantic structure of the sentences. The Stanford parser has been reported to be one of the state-of-the-art parsers in terms of speed and accuracy (7, 8). When applied to the biomedical domain, it has successfully helped to extract various types of biological relations and events from the literature (5, 29) and identify medical treatment terms from randomized clinical trial reports (3). The Stanford parser is parameterized to return both Penn Treebank parse tree and dependency representations for each sentence. While the flat version of the Penn Treebank parse tree is directly encoded into the XML, the dependency representations are recorded directly as BioC relations between participating tokens referenced by their token IDs.

Because of the Unicode compatibility of the integrated tools, the pipeline should work well over texts encoded in both ASCII and Unicode. The pipeline currently performs an end-to-end annotation from text tokenization to sentence parsing. However, even though sentence parsing is useful for tasks such as question answering or relation extraction, it is not often considered by tasks like named entity detection or concept recognition. In addition, because of the constituent-based parsing nature of the Stanford parser, sentence parsing accounts for most of the execution time of the pipeline. Options have been added, allowing the user to specify which stages in the pipeline should be run. This provides flexibility to run whichever stages are necessary and affordable, while avoiding unnecessary stages that require more time.

## Application of BioC NLP tools to the NCBI disease corpus

We used the NCBI disease corpus to provide a use case and an evaluation of the BioC NLP tools. The NCBI disease corpus (9, 10) is a manually annotated resource for disease name recognition and normalization in biomedical text, which comprises a collection of 793 PubMed abstracts and 6892 disease mentions, which further correspond to 790 disease concepts mapped to MeSH descriptors or OMIM identifiers. It was completed by a team of 14 annotators in three annotation rounds and provides a high-quality, reliable and consistently annotated resource. To make the NCBI disease corpus more accessible, and to promote its usage for other related biomedical information extraction tasks, the collection was converted to BioC format and is used here as a model test case to run the BioC NLP preprocessing pipeline tools.

**Figure 2.**
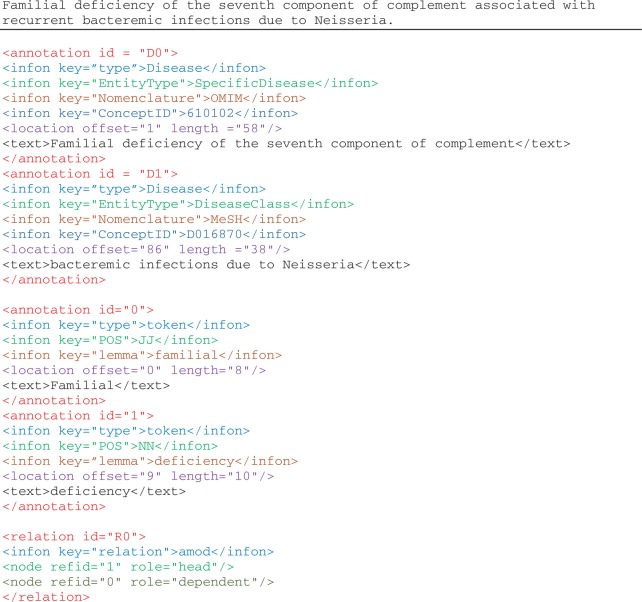
Illustration of annotations in the enriched NCBI disease corpus, manual annotations of disease mentions and concepts, and BioC-tool-produced annotations from text preprocessing.

Figure 2 illustrates the disease mention and concept annotation in the NCBI disease corpus expressed in BioC format. Each annotation contains the textual mention with the appropriate location information, given with the precise document offset and length. As in this corpus, the same textual string is annotated for both mention and concept, infons are used to express the semantics of each annotation: the infon key=“EntityType” is used to distinguish the four disease categories as specified by the annotators of the corpus, the infon key=“Nomenclature” is used to distinguish the correct terminology resource selected for the annotation and the infon key=“ConceptID” specifies the unique concept identifier for the textual mention. The infon key–value pairs in the annotation elements were produced from the original corpus format, the PubTator format (30). The tool to convert PubTator annotation data to BioC is also available for download.

We ran both C++ and Java pipelines on the BioC-formatted NCBI disease corpus, thereby enriching this resource with machine-assisted annotations and preprocessing the data ready for use by any BioC compliant application. The machine-assisted annotations consist of sentence segmentation, tokenization and POS tagging processed using both MedPost and Stanford tools, lemmatization using BioLemmatizer, parsing using both C&C and Stanford parsers, as well as abbreviation definition detection using Ab3P, Schwartz & Hearst and NatLAb algorithms (31).

## Discussion and evaluation of the NLP pipelines

### Evaluation of BioC pipelines

We ran the BioC NLP tools on the NCBI disease corpus and compared the outputs obtained in their native implementation versus the outputs obtained using their BioC-compatible counterparts. We found zero inconsistency in tokenization, sentence segmentation, POS tagging and parsing results during this evaluation exercise.

**Figure 3.**
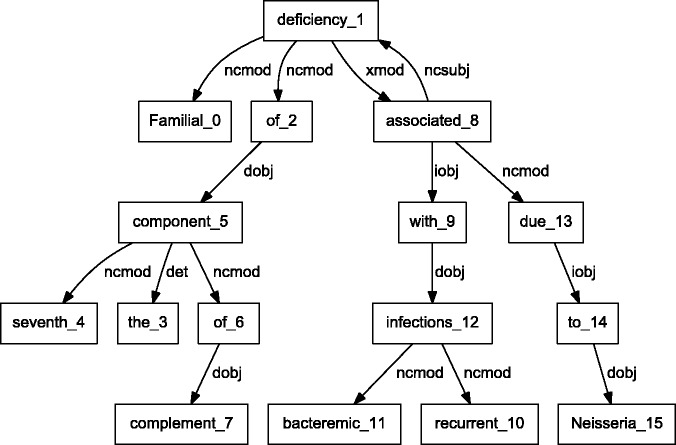
Dependency graph of ‘Familial deficiency of the seventh component of complement associated with recurrent bacteremic infections due to Neisseria’ using C&C parser.

**Figure 4.**
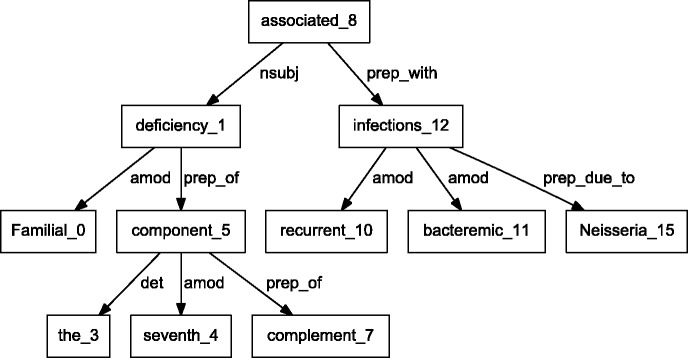
Dependency graph of ‘Familial deficiency of the seventh component of complement associated with recurrent bacteremic infections due to Neisseria’ using Stanford parser.

BioC C++ and Java NLP pipelines produce easily comparable results. We performed a detailed comparison of the outputs obtained from the two pipelines on a randomly selected set of 10 PubMed abstracts. The results showed that the segmented sentences were identical. The tokens and POS tags were similar, with only expected differences. For example, MedPost and Stanford tools make different decisions on splitting tokens containing hyphens (-) or slash (/) characters. Finally, we selected a sample sentence to illustrate the dependency parsing, and results are shown in Figures 3 and 4. The C&C dependency graph appears in Figure 3, whereas the Stanford dependency graph appears in Figure 4. The difference in the figures is expected because the parsers use different methodologies. The Stanford parser performs joint inference over the product of an unlexicalized Probabilistic Context-Free Grammar parser and a lexicalized dependency parser, although the C&C parser is based on a Combinatory Categorial Grammar (32).

By integrating output from various parsers into a unified BioC format, our pipelines allow text mining systems to take advantage of commonalities of the structural analysis of sentences. These commonalities allow the same program to produce the visual dependency graphs in Figures 3 and 4. This program reads a BioC output and prepares a graph description in the DOT language (33). This example demonstrates the value of a computer language-independent and algorithm-independent data format. An application taking advantage of parse details, such as dependency labels, would need to be tuned for a specific parser. However, dependencies can be obtained from the BioC representation of either parse.

### Advantages of language neutral format

Results in a language-neutral format can be easily combined regardless of the implementation language. An example is our technical contribution to the BioNLP Shared Task 2013. Together with other groups, we were invited by the task organizers to provide automatically generated analyses of the shared task data (12). As the Java pipeline was not available at that time, we used the BioC C++ pipeline to provide syntactic constituents and the Java-based Biolemmatizer (27) to provide lemmas for the shared task data. Through the common BioC format, these results were easily combined into a complete package of supporting materials. The data and results were made available to the public through the official shared task Web site. Eventually, three participating teams explicitly mentioned in their work that the BioC results were successfully used in their systems on different subtasks (13–15).

## Summary

We have implemented BioC natural language preprocessing pipelines in two popular programming languages: C++ and Java. They are largely based on well-known MedPost and Stanford NLP tool sets. A benefit of BioC is the interoperability between tools written in different programming languages. With these tools, it is straightforward to use the output from Stanford tools in a C++ pipeline, or the output from MedPost in a Java pipeline. We also presented the NCBI disease corpus in the BioC format. As a test case application for the pipelines, the corpus was used as input to both the C++ and Java BioC pipelines.

The BioC text preprocessing pipelines provide for automatically annotating BioC collections and can serve as starting points for more sophisticated systems. The pipelines are implemented in a flexible manner, enabling researchers to integrate other tools as needed. Alleviating the burden of interoperability challenges will encourage the exploration of novel approaches with the promise of improved text mining performance.

Our BioC pipeline was used in the BioNLP 2013 shared task on event extraction to produce preprocessing results on all subtasks. These data and code are available to the public through the BioNLP shared task Web site. The shared task data were used successfully by three separate teams.

BioC NLP pipeline tools and data are available to the community from the BioC Web site: http://bioc.source forge.net.

## Funding

This research was supported by the Intramural Research Program of the NIH, National Library of Medicine. Funding for open access charge: Intramural Research Program of the NIH, National Library of Medicine.

*Conflict of interest*. None declared.
